# Patient Preferences Regarding Device Reuse and Potential of Devices for Reuse - A Study in a Veteran Population

**DOI:** 10.1016/s0972-6292(16)30626-x

**Published:** 2013-06-25

**Authors:** Indiresha R Iyer, Judith Mackall

**Affiliations:** 1 Mount Carmel Health System, Cardiology Inc, 5969 E. Broad Street, Suite 201, Columbus OH; 2 University Hospitals, Cleveland OH, Case Western Reserve University, 11100 Euclid Avenue, Mailstop Lakeside 503B, Cleveland Oh 44106

**Keywords:** Patient preferences, Devices, Postmortem, Reuse

## Abstract

**Background:**

Many cardiac patients need and undergo device implants. Veterans' preferences regarding post-mortem handling of devices are not known. Cardiac patients in low- and middle-income countries who need but cannot afford devices rely on donations. Charitable organizations have successfully provided devices for reuse to such patients.

**Objective:**

We estimated the number of devices with potential for possible reuse in a veteran population.

**Methods:**

Between January and December 2008, at a tertiary medical center, veterans with implanted cardiac devices were surveyed using a questionnaire for their preferences regarding post-mortem handling. One choice was donation to charity for reuse. Although altruistic, it is unclear what percent of such devices have reuse potential. Retrospective chart review of veterans who underwent device implants between 1992 and 2007 identified a cohort of patients with Implantable Cardiac Defibrillators (ICDs) who had died by April 31st 2009. In this cohort, ICDs implanted in the year preceding the patient's death were counted as having reuse potential.

**Results:**

94 of 97 veterans completed the survey. 56% were unaware of how devices are handled after death. The top three preferences for postmortem handling were: return to manufacturer, return to hospital and donation for reuse. 88% were willing to sign an advance device directive. Retrospective review identified 161 veterans who had received 301 ICDs. Of these, 77 ICDs (25%) had median reuse potential of 3.1 years.

**Conclusion:**

In a VA cohort of deceased patients a substantial proportion of devices had reuse potential. Further research is needed to direct health policy.

## Introduction

Sudden cardiac death (SCD) and heart failure utilize significant health care resources.[1] Device therapy for SCD and heart failure [2-6] have decreased mortality and patients tend to live longer.[7] While the number of Implantable Cardioverter Defibrillator (ICD) implantations in the US has increased, [8] global inequalities in health care access result in low device utilization rates in low- and middle-income countries [9] despite the growing burden of cardiovascular disease.

Charitable organizations have been providing devices, generously donated by industry or harvested by physicians, to patients in low- and middle-income countries.[10,11] Recently published studies have shown that there is no increase in morbidity or mortality associated with device reuse [19]. Large scale clinical studies of device reutilization in underserved countries have been suggested [12] but the availability of devices with potential for reuse is unknown.

In a survey, 10% of responding electrophysiologists had harvested devices for purposes of donation [13]. The HRS guidelines recommend that physicians seek patients' consent for post-mortem device retrieval while they are alive.[14] In routine practice, informed consent for device implants rarely includes a discussion regarding post-mortem handling of devices. A survey of patient preferences showed that 87% of 150 patients did not know how devices were handled after their death. The majority were willing to have their devices retrieved for return to the manufacturer or for reuse.[15]

Patient preferences for post-mortem handling affect health policy. We studied these preferences in a veteran population. We also estimated the availability of devices with potential for reuse in another veteran cohort from the same institution. Such data affect the feasibility of larger scale clinical studies.

## Methods

The first protocol for patient preferences consisted of a confidential close ended survey administered to a sample of patients with cardiac implanted devices (implantable cardiac defibrillators) who attend the device clinic at the Louis Stokes Cleveland Department of VA Medical Center (LSCDVAMC). The survey was a structured questionnaire of patient preferences regarding post-mortem handling of devices. The survey was based on the prior work of Kirkpatrick et al [15] and Wild et al [16]. The questionnaire was provided to patients consenting to participate in the survey. After their clinic visit, patients independently completed the survey, sometimes with help from their caregiver. The investigator answered additional questions after completion of the survey to minimize biasing patients' choices. The questionnaire did not collect any patient identifiers.

Data collected included age, height, weight, type of device, years since their first device implant and the number of device implants. The survey asked patients if they had a living will, health care proxy or designated power of attorney followed by their idea of how devices were handled after their death. Patients then ranked 8 possible choices for post-mortem handling of devices including donation for reuse (Table 1). They were asked if they were willing to sign a directive regarding their choices for postmortem handling.

A second protocol was used to estimate the number of ICDs with potential for reuse. Some of the data from this protocol has been published [17]. The charts of all patients who underwent device implants at LSCDVAMC between June 1992 and April 2007 were reviewed. Information regarding the device, procedure, complications, follow-up and subsequent procedures was obtained. The Social Security death index and the charts were used to identify a cohort of these patients who were no longer alive as of April 31st 2009. Detailed device interrogation data was not available for many of these patients.

In this cohort, two sets of devices were identified. The first set consisted of all ICD/CRT-Ds that were replaced for battery depletion alone over the life of the patients. From this set, "actual longevity" was derived. The "actual longevity" was defined as the number of days the devices had lasted before replacement for normal battery depletion. For each device this was calculated as the number of days from Date of Implant to Date of Replacement.

The second set consisted of the ICD/CRT-Ds in the patient at the time of their death. From this set, devices with "reuse potential" were identified. These were devices that had been implanted within 1 year preceding the patients" death. For these devices with reuse potential, "utilized longevity" was calculated as the number of days from Date of Implant to Date of Death. The"magnitude of reuse potential" was defined as the difference between the Median "actual longevity" of all devices replaced for normal battery depletion (set 1) and the Median "utilized longevity" (set 2).

Since we used the median duration that devices lasted in the first set and the median duration of device use in the second set, we feel that this method of measuring the "magnitude of reuse potential" is a valid measure of expected remaining battery longevity. The protocols were approved by the IRB.

### Statistical Methods

The data were tabulated in Microsoft Excel and analyzed using SPSS 15.0. Descriptive statistics are reported for the survey. BMI was dichotomized into ≥ 25.0 and < 25.0 kg/m^2^.

## Results

Of 97 patients who were asked to participate in the survey, 94 patients completed the survey for a response rate of 97%. One patient filled out part of the survey. Two additional patients refused. The mean age was 67.7 years. Only two patients were younger than 50 years. There was a single female in this group. The median Body Mass Index (BMI) was 30.5. Sixty nine (73.4%) patients were overweight or obese. The majority of patients had undergone their implant more than a year ago. The currently active device was the first for 74 patients. The type of device distribution is shown in Table 2.

### Survey Results

*Disposition after death:* The majority (53 patients or 56.4%) of patients did not have any idea of what happened to their device post mortem. 16, 12 and 10 patients respectively believed that the device was sent back to the company, buried with them, or reused/recycled while 3 patients thought it would be donated to charity.

*Preferences for Post mortem Handling:* Seventy two patients (76.5%) chose at least one of the listed options for post mortem handling of their device and rank ordered their preferences. One interesting observation was that no one wanted to be buried with their device including the twelve patients who thought that would be the case. Every patient except one, expressed strong feelings about not wanting the device to be buried with them.

The majority of patients (61.7% or 58 patients) had a living will, health care proxy or a durable health care power of attorney. In this group, 83 patients (88%) were willing to state their preferences for post-mortem handling of their device in writing in the form of a device directive.

### Identifying ICDs with "reuse potential"

A total of 379 patients underwent 646 ICD/CRT-D device implants, between August 1992 and April 2007. Of these, 161 patients had died by April 2009. These 161 patients had received a total of 301 ICD implants of which 147 ICDs were replaced for normal battery depletion in 61 patients. These 147 ICDs with normal battery depletion were replaced after a median of 1295 days (median "actual longevity"). Among the patients who died, there were 77 devices that had been implanted less than a year prior to the patient's death (median "utilized longevity" 161 days). This gives an estimated magnitude of "reuse potential" of 1134 days for the 77 devices. The cause of death was unrelated to the ICD implant procedure. These 77 devices with median "reuse potential" of 3.1 years constituted 25.6% of the ICDs in this cohort. Additionally in 3 patients, ICDs were replaced within a month due to high DFTs and 2 other patients were upgraded to CRT-Ds within 6 months of ICD implant. These 5 devices if included would result in 82 devices with significant reuse potential.

## Discussion

### Patient preferences

Very few patients have any idea of the way devices are handled post mortem. This finding is similar to that of Kirkpatrick et al.[15] The vast majority of patients (99%) in this survey did not want to be buried with their device. The top 3 patients' preferences were donation to charity, return to the manufacturer, and return to the hospital. This is aligned with the Heart Rhythm Society task force guidelines which strongly encourages postmortem device interrogation, explantation and return to the manufacturer particularly in cases of sudden or unexpected death.[14]

Return of retrieved devices to manufacturers or hospitals would increase the number of devices available for post mortem returned product analysis. Bartsch C et al have shown that there is much undetected device dysfunction in their post mortem analysis of 415 pacemakers (and ICD) by in-situ and bench tests. Their postmortem evaluated rate of dysfunction in the life-threatening category of 3.8% and premature exhaustion of battery of 1.2% corresponded to an annual complication rate of 0.94%. The annual ICD malfunction replacement rate was significantly higher than the pacemaker malfunction replacement rate (mean [SD], 20.7 [11.6] vs. 4.6 [2.2] replacements per 1000 implants.[18]

In our survey, 44.6% of patients wished to donate their devices for post mortem reuse as their first choice and 79% chose this among their top three options. 88% were willing to sign a device directive. In the survey of Kirkpatrick et al 91% of patients chose this option.[15]

Practical limitations for this preference include communicating this preference to the mortician and sending the explanted device to an organization that can test the device for useful battery life, test for normal function, clean/sterilize it and then send it to an appropriate institution for implantation in a deserving recipient in a country where it is considered legal and ethical.

### Reuse potential of explanted devices

The analysis of the VA database showed that 25.6% of all devices implanted in the deceased cohort had potentially useful remaining battery life. If reused in economically disadvantaged individuals in low- and middle- income countries with an ICD indication for secondary prevention of SCD, the median reuse potential of 1134 days (3.3 years) is the duration for which 77 patients would receive some protection from SCD.

At least 25% of these patients will receive appropriate therapy over a year based on the results of trials of secondary prevention. This is in concordance with a recently published study from India, in which explanted ICDs with at least 3 years of remaining battery life were safely reused after careful cleaning and resterilization. These ICDs functioned appropriately in delivering life-saving therapies without an increased risk of complications.[19]

Reuse of devices poses risks such as infection, device malfunction or premature battery depletion. In Sweden pacemakers had been successfully reused in the 1990s for several years. In one retrospective study, 100 patients who received reused pacemakers were compared to 100 patients who received a new pacemaker and followed for a mean of 32 +/- 11 months. There were no early replacements due to battery depletion in either of the groups and a cost benefit analysis showed cost savings. Reuse without increased risk was feasible as long as proper technical control and sterilization protocols were followed.[20]

Baman et al have shown that it is possible to successfully harvest devices with remaining useful battery life and reuse them in patients in low- to middle- income countries. In their study, 18 of 50 donated pacemakers from Detroit metropolitan area funeral homes to World Medical Relief had 70% battery life. 12 patients in the Philippines underwent implantation and suffered no complications over a 2- month follow up period.[21]

Hasan et al published a case series of 17 Nicaraguan patients with a Class 1 indication for a Cardiac Implantable Device who received devices that were harvested ante mortem from patients at a single US institution. During the long term follow up of 68 +/- 38 months, there were no infections, device malfunction or premature battery depletion. One patient had a hematoma at the time of implant.[22] A meta-analysis of over 600 patients in 4 clinical trials did not show any evidence of increased infection risk with reused pacemakers compared to new implants.[23] These data suggest that a significant proportion of devices may have the potential for reuse. It may be reasonable to support larger studies to better address these risks.

The incidence of cardiovascular disease is projected to increase by 137% between 1990 and 2020 in Low- and Middle- Income countries while financial constraints continue to increase the disparity in health services between industrialized nations and Low- and Middle- income countries, especially in the field of devices.[24] Device re-utilization may be an option for such patients. Considerable organization and collaboration between hospital systems in different countries, funeral homes and charitable organizations is necessary for such reuse. Organizations such as World Medical Relief [11] have protocols to reuse explanted devices and have provided donated pacemakers for reuse successfully although there are several logistical, ethical and legal barriers.[25,26] An initiative to reuse devices to alleviate symptomatic bradyarrhythmia in low- and middle- income countries has been proposed.[27]

Incorporating patient preferences has significant implications for patient safety, health policy, health care economics and the device industry.

## Limitations

This is a small sample confined to a specific patient population in one geographic region and may not be applicable to other populations. The data regarding the number of devices with potentially usable battery life was derived from a retrospective analysis of patients' whose devices and battery technology are different from currently used devices; recent generation devices show longer battery life, and therefore may offer greater reuse potential. This study also does not take into account any patient factors and actual remaining battery power was not measured. The majority of patients in this cohort had a secondary indication for ICD placement. These reasons would affect the magnitude of "reuse potential" although the methodology used here is more likely to have underestimated remaining device longevity.

A study utilizing large datasets maintained by device manufacturers in conjunction with multiple academic institutions may give a better estimate of number of devices with significant reuse potential. Further research is needed to direct policy.

## Conclusion

Most patients are unaware of how devices are handled after their death. A majority of patients would express their preference in the form of a written directive. Patient preferences are aligned with the Heart Rhythm Society's recommendations for morticians to harvest devices and return it to the manufacturer for device testing. Patients are willing to donate their devices for other uses after their death. A significant proportion of devices at the time of death may have potential for reuse.

## Figures and Tables

**Table 1:**
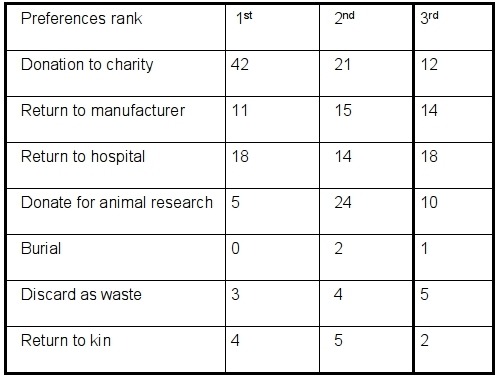
Patient preferences for post-mortem handling of devices

**Table 2:**
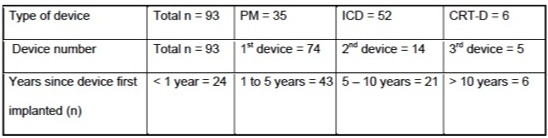
Device characteristics
